# Prospective evaluation of core number of biopsy for renal tumor: are multiple cores preferable?

**DOI:** 10.1007/s11604-023-01496-x

**Published:** 2023-10-14

**Authors:** Toshihiro Iguchi, Yusuke Matsui, Tomohiro Toji, Jun Sakurai, Koji Tomita, Mayu Uka, Noriyuki Umakoshi, Takahiro Kawabata, Kazuaki Munetomo, Toshiharu Mitsuhashi, Takao Hiraki

**Affiliations:** 1 Department of Radiology, Okayama University Hospital, 2-5-1 Shikata-cho Kita-ku, Okayama, 700-8558 Japan; 2 Department of Radiological Technology, Faculty of Health Sciences, Okayama University, 2-5-1 Shikata-cho Kita-ku, Okayama, 700-8558 Japan; 3 Department of Radiology, Faculty of Medicine, Dentistry and Pharmaceutical Sciences, Okayama University, 2-5-1 Shikata-cho, Kita-ku, Okayama, 700-8558 Japan; 4 Department of Pathology, Okayama University Graduate School of Medicine, Dentistry, and Pharmaceutical Sciences, 2-5-1 Shikata-cho Kita-ku, Okayama, 700-8558 Japan; 5 Center for Innovative Clinical Medicine, Okayama University Hospital, 2-5-1 Shikata-cho Kita-ku, Okayama, 700-8558 Japan

**Keywords:** Biopsy, Kidney, Tumor, Computed tomography, Ultrasound

## Abstract

**Purpose:**

This single-center, single-arm, prospective, open-label study was conducted to evaluate the optimal number of cores (single or multiple) in renal tumor biopsy.

**Materials and methods:**

Forty-four biopsies of 44 tumors (mean diameter, 2.7 ± 1.0 cm; range, 1.6–5.0 cm) were included. Biopsy was performed under ultrasound or computed tomography fluoroscopy guidance using an 18-gauge cutting needle and the co-axial method. Two or more specimens were obtained, which were divided into first and subsequent specimens. “First specimen” and “all specimens” were histologically evaluated (i.e., appropriateness of specimen, histological diagnosis, subtype, and Fuhrman grade of renal cell carcinoma [RCC]) blindly and independently by two board-certified pathologists.

**Results:**

Multiple specimens were successfully and safely obtained in all the biopsies. All tumors were histologically diagnosed; 40 malignancies included 39 RCCs and 1 solitary fibrous tumor, and 4 benign lesions included 2 angiomyolipomas, 1 oncocytoma, and 1 capillary hemangioma. In all RCCs, the subtype could be determined (32 clear cell RCCs, 4 chromophobe RCCs, and 3 papillary RCCs), and the Furman grade was determined in 38 RCCs. When only the first specimen was evaluated, 22.7% of the specimens were inappropriate for diagnosis, and 34 (77.3%) were histologically diagnosed. The diagnostic yield was significantly lower than that of all specimens (*P* = 0.0044). Univariate analysis revealed that smaller lesions were a significant predictor of diagnostic failure (*P* = 0.020).

**Conclusion:**

Biopsy with multiple cores significantly improved diagnostic yield. Thus, operators should obtain multiple cores during renal tumor biopsy.

## Introduction

The increased opportunity for imaging examinations (e.g., ultrasound [US], computed tomography [CT], and magnetic resonance imaging) increases the incidental detection of renal tumors in asymptomatic patients [1]. Except for some types of typical benign lesions (e.g., angiomyolipoma and Bosniak I or II cysts), renal tumors are difficult to correctly diagnose using imaging alone. In such situations, a percutaneous image-guided biopsy may be performed. This procedure was previously avoided because of the risk of complications (bleeding and tumor seeding) and false-negative diagnoses. However, recent results have attested to its safety and high diagnostic yields [2–7], and biopsy is strongly recommended before ablative and systemic therapies without previous pathology [8].

Guidelines and international consensus panels have technical recommendations (e.g., number of cores, puncture site, and biopsy needle size) to achieve diagnostic success in image-guided biopsy for renal tumors. Although the ideal number of cores has not been defined, some guidelines have suggested at least two good-quality cores [8, 9]. A larger number of cores facilitates the collection of a larger amount of tissue, and the diagnostic yields are expected to be higher with multiple cores than with a single core. However, little evidence supports the significance of the core number (“two or more”) in biopsies of renal tumors. Therefore, robust clinical data are required to conclude that the desired number of cores is at least two. We hypothesized that the diagnostic yield of a multiple-core biopsy would be higher than that of a single-core biopsy. This study prospectively evaluated the optimal number of cores (single or multiple) for renal tumor biopsies.

## Materials and methods

### Study design

This single-center, single-arm, prospective, open-label study was conducted between October 2020 and April 2022, with approval from the Institutional Review Board (approval number: RIN2009-005) and in accordance with the Declaration of Helsinki. All participants provided written informed consent. This study was registered in the University Hospital Medical Information Network Clinical Trials Registry (study ID: UMIN000041358).

### Study end points

The primary end point was the diagnostic yield of single-core and multiple-core biopsies. The secondary end points were i) feasibility of multiple-core biopsy, ii) appropriateness of specimens obtained by single-core and multiple-core biopsies, iii) histological diagnosis, iv) renal cell carcinoma (RCC) subtype, and v) Fuhrman grade of RCC.

### Eligibility criteria

The inclusion criteria were i) renal tumor with an indication for image-guided biopsy, ii) tumor > 1.5 cm, and iii) age ≥ 20 years. The exclusion criteria were i) cystic tumor; ii) tumor with an obvious fat component (i.e., typical angiomyolipoma); iii) abnormal laboratory data, including white blood cell count < 2,000/μL, hemoglobin level < 6.0 g/dL, platelet count < 50,000/μL, and prothrombin time > 1.5; iv) pregnancy; v) mental illness (e.g., dementia); and vi) ineligibility determined by the responding physician.

### Biopsy procedure

All biopsies were performed in the interventional radiology suite under US (Aplio 500; Canon Medical Systems, Otawara, Japan) or CT fluoroscopy (Aquilion; Canon Medical Systems) guidance. An operator completed the biopsies with the co-axial method using one image guidance modality per procedure. The co-axial system comprised a 17-gauge introducer and an 18-gauge semi-automatic cutting biopsy needle (Temno Evolution, Care Fusion, IL, USA). A board-certified interventional radiologist (staff) or non-staff member, under a staff member’s direct supervision, performed the biopsy. Operators included four staff with 15 years of median experience (range, 10–16 years) and two non-staff with 6 and 3 years of experience, respectively.

First, the patient was placed on a CT table under conscious sedation, and an US examination was performed to detect the target renal tumor. If possible, biopsy was performed under US guidance. If the target was undetected with US or CT guidance appeared safer and/or more reliable, the biopsy was performed under CT fluoroscopy guidance. Before CT fluoroscopy-guided biopsy, a conventional abdominal CT examination with a 5-mm slice thickness was performed to precisely locate the tumor and plan the needle insertion route. If the target was not visible on plain CT images, a contrast medium was administered intravenously for tumor visualization before obtaining the first specimen. After administration of local anesthesia with lidocaine, the introducer needle was advanced until its tip was in front of the tumor. The internal stylet of the introducer needle was replaced with a biopsy needle. Furthermore, multiple specimens were obtained until the quantity was sufficient (e.g., a total core length > 2 cm). When operators lacked certainty regarding needle placement within the targeted tumor, additional biopsies were performed until they were certain that the target had been successfully biopsied. The tumor target area (i.e., central or peripheral) for the biopsy was determined at the operator’s discretion. Biopsy specimens were divided into first and subsequent specimens and were diagnosed histopathologically. Complications were evaluated using US or plain conventional CT with a ≤ 5-mm slice thickness. All complications were graded according to the Clavien–Dindo classification of surgical complications [10].

### Histological evaluation

**Fig. 1:**
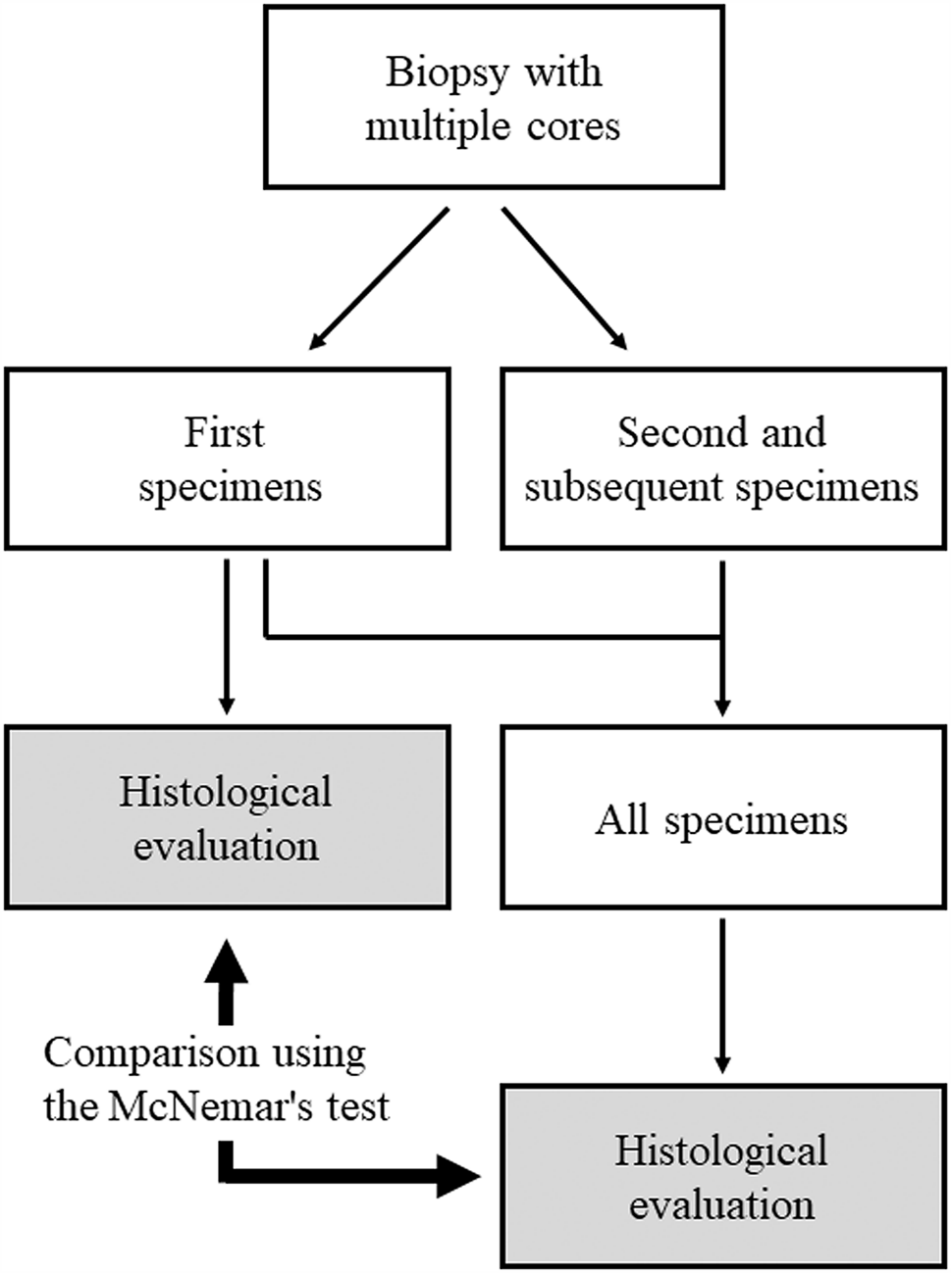
Flowchart of histological evaluation

The specimens were evaluated for their appropriateness for diagnosis. The pathologists evaluated and diagnosed the benign or malignant tumors histologically. In RCC tumors, the subtype and Fuhrman grade were evaluated. The first and subsequent specimens were paraffin-embedded separately, and tissue specimens were prepared. Evaluations of “the first specimen” and “all specimens” were blindly and independently performed by two board-certified pathologists with 31 and 16 years of experience (other and T.T.); the diagnosis was made via consensus (Fig. 1). If a benign lesion was histologically diagnosed, a 3-month imaging follow-up was performed to confirm whether the tumor size was unchanged.

### Sample size calculation

According a previous study’s results [11] (59% diagnostic yield with one core, 77–80% with two cores [vs. one core, *P* < 0.01], and 85% with three cores [vs. one core, *P* = 0.001]), we set the diagnostic yield of the first specimen = 0.59 and that of all specimens (i.e., first and subsequent specimens) = 0.80. Furthermore, we set *α* = 0.05 and *β* = 0.2. Because this study compares the diagnostic yield between the first specimen and all specimens for the same patient, we calculated a sample size sufficient to detect differences using McNemar's test.

The probabilities of a 2 × 2 table under these conditions are as follows (Table 1**)**. The probability of “b” = 0 because diagnosis by all specimens was always possible when diagnosis by the first specimen was possible. The formula had to be assigned a small value instead of *b* = 0 for the calculations. Using *b* = 0.01 to 10^–10^ as a small number, 35–41 pairs were needed. Since the pairs are “the first specimen” and “all specimens,” the required tumor number was between 35 and 41. Calculating where the actual number in cell b is 0 pairs and the actual number in cell c is six pairs or more, McNemar’s test showed significance (“a” and “d” do not contribute to the test results). This number increased by 10% in anticipation of protocol deviations. Therefore, we enrolled 45 patients with renal tumors.

**Table 1:** Probability of 2 × 2 table

		Diagnosis of all specimens		
		Possible	Impossible	
Diagnosis of first specimen	Possible	0.59 (a)	0 (b)	0.59
	Impossible	0.21 (c)	0.2 (d)	0.41
		0.8	0.2	1

### Statistical analysis

The diagnostic yields of the first specimen and all specimens were calculated and compared using McNemar’s test. The core number was compared between the two groups (i.e., biopsies with and without complications) using the Mann–Whitney *U* test.

The histological results of the first specimen were classified as a diagnostic success or failure. Patient-, tumor-, and biopsy-related variables were evaluated to assess risk factors for diagnostic failure. Age and sex were included as patient-related variables. Tumor-related variables included size, laterality (left or right kidney), anteroposterior location (ventral or dorsal), longitudinal location (upper or lower pole), position (exophytic or non-exophytic), and diagnosis (benign lesion or malignancy). Biopsy-related variables included operator experience (staff or non-staff), guided image modality (CT or US), and tumor puncture site (central or non-central). Tumor position was classified as exophytic or non-exophytic (parenchymal, mixed, and central) according to Gervais et al.’s definition [12]. Tumor locations were classified according to the transverse and longitudinal kidney axes as follows: ventral (tumor arises from the anterior half), dorsal (tumor arises from the posterior half), upper pole (tumor arises from the upper half), and lower pole (tumor arises from the lower half).

The variables were compared between the two groups using Fisher’s exact test for categorical values and the Mann–Whitney *U* test for numerical values. Two authors (J.S. and K.T.) performed analyses using R version 3.6.1 and SPSS version 26 (IBM Corp., Armonk, NY, USA). Statistical significance was set at *P* < 0.05.

## Results

**Fig. 2:**
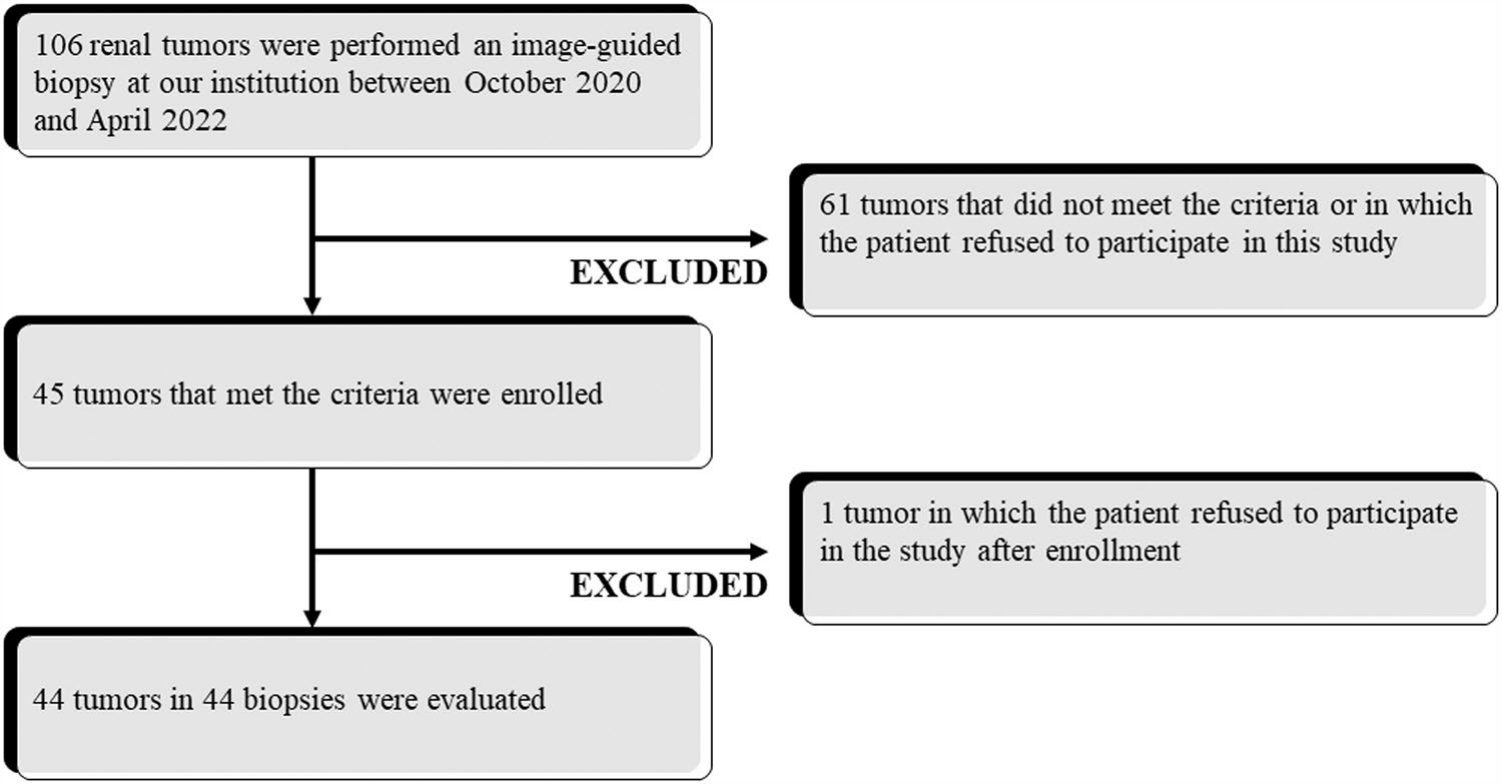
Flowchart diagram with the number of tumors

The characteristics of the patients, tumors, and biopsy procedures are summarized in Table 2. Forty-five renal tumors in 45 patients were enrolled. One patient withdrew from the study immediately before the biopsy procedure. Therefore, this study included 44 renal tumors (mean diameter, 2.7 ± 1.0 cm; median diameter, 2.45 cm; range, 1.6–5.0 cm) in 44 patients (29 men and 15 women; mean age, 68.0 ± 11.6 years; range, 37–86 years) (Fig. 2). In six tumors, the diameter was larger than 4.0 cm. Thirty tumors were exophytic, 5 were parenchymal, 7 were mixed, and 2 were central. The anteroposterior tumor locations were ventral and dorsal in 24 and 20 tumors, respectively. The longitudinal tumor locations were the upper and lower poles in 21 and 23 tumors, respectively. One tumor included in this study has been previously published as a Japanese case report [13], which was imaged by four-dimensional CT.

**Table 2:** Characteristics of 44 patients, 44 tumors, and 44 biopsies

Variable		Value
Patient		
Age (y)	Mean ± SD (range)	68.0 ± 11.6 (37–86)
Sex	Man/woman	29/15
Tumor		
Size (cm)	Mean ± SD (range)	2.7 ± 1.0 (1.6–5.0)
Laterality	Left/right	22/22
Antero-posterior location	Ventral/dorsal	24/20
Longitudinal location	Upper/lower	21/23
Position	Exophytic/parenchymal/mixed/central	30/5/7/2
Biopsy		
Patients positioning	Prone/supine	44/0
Operator experience	Staff/non-staff	30/14
Guiding modality	Unenhanced CT/enhanced CT/ultrasound	19/12/13
Number of cores	Mean ± SD (range)	4.0 ± 0.8 (3–6)
Complication	Yes/no	28/16

*SD* standard deviation, *CT* computed tomography

For the 44 renal tumors, multiple biopsy cores were obtained by 30 staff and 14 non-staff. The mean core number was 4.0 ± 0.8 (range, 3–6). US guidance (*n* = 13) and CT fluoroscopy with (*n* = 19) or without (*n* = 12) contrast medium guidance were used. Complications occurred in 28 (28/44; 63.6%) biopsies, all of which had grade 1 bleeding. No significant difference was observed in the core number between the two groups (28 biopsies with [3.89 ± 0.74] and 16 without complications [4.06 ± 0.85]; *P* = 0.58).

All the 44 renal tumors, including 4 benign and 40 malignant lesions, were histologically diagnosed using all specimens. Four benign lesions included 2 angiomyolipomas, 1 oncocytoma, and 1 capillary hemangioma, whereas 40 malignancies included 39 RCCs and 1 solitary fibrous tumor (Table 3). The subtypes of all 39 RCCs could be determined (i.e., 32 clear cell RCCs, 4 chromophobe RCCs, and 3 papillary RCCs), and the Furman grade of 38 RCCs (38/39; 97.4%) could be determined (Table 3).

**Table 3:** Histological diagnosis by evaluating all specimens

	Diagnosis	Furman grade of RCC					
		Subtype of RCC	1	2	3	NA	Number
Malignancy	RCC	Clear cell	17	13	1	1	32
		Chromophobe		4			4
		Papillary	1	2			3
	Solitary fibrous tumor						1
Benign lesion	Angiomyolipoma						2
	Oncocytoma						1
	Capillary hemangioma						1

*RCC* renal cell carcinoma, *NA* not applicable

When only the first specimen was evaluated, 10 specimens (10/44; 22.7%) were inappropriate for diagnosis, 34 tumors (34/44; 77.3%) were histologically diagnosed, and the diagnostic yield using only the first specimen was significantly lower than that using all specimens (*P* = 0.0044; Table 4). Of the ten tumors with diagnostic failure, three could not be diagnosed because of the small specimen volume, and the other results were renal parenchyma (*n* = 3), blood (*n* = 2), fat (*n* = 1), and fibrous tissue (*n* = 1). The final diagnoses included eight clear cell RCCs (five Furman grade 1 and three grade 2), one chromophobe RCC (grade 2), and one angiomyolipoma. Univariate analysis revealed that smaller lesions were a significant predictor of diagnostic failure (*P* = 0.028; Table 5).

**Table 4:** Histological results by evaluating first and all specimens in 44 biopsies

		Diagnosis by evaluating all specimens			
		Benign lesion	Malignancy	Diagnostic failure	Total
Diagnosis by evaluating first specimen	Benign lesion	3	0	0	3
	Malignancy	0	31	0	31
	Diagnostic failure	1	9	0	10
	Total	4	40	0	40

**Table 5:** Variables in diagnostic success and failure groups in 44 first specimens

Variable		Diagnostic success (*n* = 34)	Diagnostic failure (*n* = 10)	*P* value
Patient				
Age (y)	Mean ± SD (range)	68.5 ± 11.1 (44–86)	66.0 ± 13.4 (37–80)	0.689
Sex	Man/woman	24/10	5/5	0.271
Tumor				
Size (cm)	Mean ± SD (range)	2.9 ± 1.1 (1.6–5.0)	2.1 ± 0.5 (1.6–3.0)	0.028
Laterality	Left/right	17/17	5/7	> 0.99
Antero-posterior location	Ventral/dorsal	19/15	5/5	> 0.99
Longitudinal location	Upper/lower	18/16	3/7	0.287
Position	Exophytic/non-exophytic	23/11	7/3	> 0.99
Diagnosis	Benign lesion/malignancy	3/31	1/9	> 0.99
Biopsy				
Operator experience	Staff/non-staff	25/9	5/5	0.247
Guiding modality	CT/ultrasound	23/11	8/2	0.697
Puncture site	Central/non-central	21/13	4/6	0.287

*SD* standard deviation, *CT* computed tomography

## Discussion

This prospective study showed that all 44 renal tumors could be histologically diagnosed by image-guided cutting needle biopsy with multiple cores without major complications. However, the diagnostic yield was significantly lower (34/44, 77.3%) when only the first specimen was evaluated. Our results may encourage operators to perform renal tumor biopsies with multiple cores rather than a single core.

Image-guided biopsy for renal tumors is widely known as a safe procedure with high diagnostic yields. One meta-analysis including 17 studies on core biopsies showed that the rate of non-diagnostic biopsies was 0–22.6%; the estimates for the sensitivity and specificity of diagnostic core biopsies based on bivariate analysis were 99.1% and 99.7%, respectively [14]. Several risk factors for non-diagnostic biopsies have been reported, including non-exophytic tumors [3], smaller tumors [3, 15, 16], cystic features [15, 16], upper pole tumors [16], thinner needle size [17], and single fire [11]. Considering these risk factors, a small diameter (≤ 1.5 cm) and cystic tumors were excluded from our study, and operators always used an 18-gauge cutting needle. The diagnostic yield of multiple specimens was 100%.

The optimal core number to be taken in renal tumor biopsies has not been defined [9]. In the clinical setting, operators usually perform biopsies with multiple cores; in many studies, the core number used was two or more [4–6, 18]. Although an ex vivo prospective study with 48 resected renal tumors (median, 4.2 cm) suggested that increasing the number of cores improves the diagnostic yield [11], no clinical results have prospectively evaluated the core number.

We believe that the co-axial method should be used to perform biopsies with multiple cores. This method has been suggested to improve diagnostic yield and increase standardization of tissue samples [19] and may be viewed as an aid in preventing tumor seeding. Additionally, the co-axial method may reduce the procedure time because needle repositioning is not required after each core.

The diagnostic yield of a biopsy with only one core is unknown. Our relatively low diagnostic yield (34/44; 77.3%) seems to be unacceptable compared with the recently favorable results of renal biopsies (> 90% diagnostic yield). In the evaluation using only the first specimen, the lesion diameter was the sole risk factor for diagnostic failure and is already a well-known predictor. This result suggests that obtaining multiple specimens is necessary, especially for smaller tumors (e.g., < 2 cm). For larger tumors, a biopsy of the tumor’s peripheral region is preferred to avoid central necrosis [20]; however, our results showed no differences in diagnostic yield by puncture site (central or non-central), probably because the tumor diameter was not large.

Investigators have reported that the core number is a factor affecting diagnostic yields in other organs. Wu et al. reported that in a prospective study with 151 consecutive image-guided core-needle biopsies of bone (*n* = 88) and soft-tissue (*n* = 63) lesions, the diagnostic yield increased with the number of specimens obtained and reached a plateau at three specimens for bone lesions and four specimens for soft-tissue lesions [21]. Fishman et al. reported that in 73 consecutive biopsies of breast masses, cells indicating the diagnosis were contained in the first specimen in 51 lesions (70%), in the second specimen in 67 (92%), in the third specimen in 70 (96%), and in the fourth specimen in 73 (100%) [22]. Although obtaining more than a certain number of specimens may not be required, multiple specimens may be preferable over a single specimen.

In image-guided biopsy for renal tumors, several complications, such as bleeding, infection, pneumothorax, arteriovenous fistula, and dissemination, have been reported. The most common complication is bleeding (e.g., hematoma and hematuria). As the core number increases, the frequency of such complications also increases. However, complications often occur in this procedure, most of which are minor and asymptomatic, and major complications are uncommon [14]. Complicated bleeding with some treatments is unusual (0–1.4%) and generally self-limiting [14]. Therefore, obtaining multiple specimens may benefit patients by increasing diagnostic yields rather than increasing the risk of minor complications.

This single-institution prospective study had some limitations. First, only the 18-gauge biopsy needle was used. Second, the diagnostic yield was not compared between one core and two or three cores. Third, no significant relationship existed between the occurrence of complications and the core number. However, as this study was not specifically designed to evaluate it, the effect of multiple-core biopsies on complications remains uncertain. Fourth, the first specimen was evaluated, followed by all specimens, including the first specimen. Therefore, we cannot definitively conclude that the assessment of the first specimen did not influence the evaluation of all specimens. Finally, we did not set an upper limit for the number of biopsies samples. The study was conducted in accordance with the clinical setting so that patients would not have any disadvantages. Therefore, the diagnostic yields of biopsies with multiple cores and those with a single core were compared. In future, it would be interesting to compare the diagnostic yield between one core and two or three cores.

In conclusion, a biopsy with multiple cores could significantly improve the diagnostic yield compared with a biopsy with only one core. Thus, operators should obtain multiple cores during renal tumor biopsy.
